# Hyperhomocysteinaemia in rats is associated with erectile dysfunction by impairing endothelial nitric oxide synthase activity

**DOI:** 10.1038/srep26647

**Published:** 2016-05-25

**Authors:** Weijun Jiang, Lei Xiong, Bin Yang, Weiwei Li, Jing Zhang, Qing Zhou, Qiuyue Wu, Tianfu Li, Cui Zhang, Mingchao Zhang, Xinyi Xia

**Affiliations:** 1 Department of Reproduction and Genetics, Institute of Laboratory Medicine, Jinling Hospital, Nanjing University School of Medicine, Nanjing 210002, P.R. China; 2 Department of Cardiothoracic surgery, Jinling Hospital, Nanjing University School of Medicine, Nanjing 210002, P.R. China

## Abstract

To investigate the effect of hyperhomocysteinaemia (HHCy) on penile erectile function in a rat model, a methionine-rich diet was used in which erectile function, the reproductive system, and nitric oxide synthase were characterized. The intracavernous pressure, apomorphine experiments, measurement of oxidative stress, hematoxylin and eosin staining, immunohistochemistry analysis, reverse transcription-polymerase chain reactions and measurement of endothelial nitric oxide synthase activity were utilized. Our results showed that erections in the middle-dose, high-dose, and interference (INF) groups were significantly lower than the control (*P* < 0.05). INF group, being fed with vitamins B and folic acid, demonstrated markedly improved penile erections compared with the middle-dose group (*P* < 0.05). HHCy-induced eNOS and phospho-eNOS protein expression was reduced and the antioxidant effect was markedly impaired. The data of the present data provide evidence that HHCy is a vascular risk factor for erectile dysfunction by impairing cavernosa endothelial nitric oxide synthase activity. Intake of vitamins B can alleviate this abnormality.

Recently, several epidemiologic studies have shown a high incidence and prevalence of penile erectile dysfunction (ED) worldwide, affecting up to 150 million men^1^,^2^,^3^. In the Massachusetts Male Aging Study, a large community-based observational survey of men 40–70 years of age, the prevalence was 52% and graded as minimal in 17.2%, moderate in 25.2%, and complete in 9.6%^4^. Penile erection is a series of complicated and coordinated physiologic activities, such as neuroendocrine and hemodynamic changes, in which many factors interact with nerve vascular phenomena. Risk factors for cardiovascular disease (CVD), including hypercholesterolemia, hypertension, diabetes mellitus (DM), and smoking are also harmful to erectile function because the penis is a richly vascularized organ^2^. Endothelial dysfunction is a common feature of these cardiovascular risk factors, and is considered to be pivotal in the association with ED^2^,^5^. These findings suggest that ED is another manifestation of systemic vascular disorder.

Another risk factor for endothelial dysfunction and CVD is an elevation of the amino acid, homocysteine (i.e., hyperhomocysteinaemia [HHCy]). Homocysteine (HCy) is generated from methionine (Met) through the successive enzymatic activations of S-adenosylmethionine and S-adenosylhomocysteine. Once generated, S-adenosylmethionine and S-adenosylhomocysteine are metabolized by cystathionine b-synthase (CBS) to cysteine via the trans-sulfuration pathway or remethylated to Met by 5, 10-methylenetetrahydrofolate reductase or betaine homocysteine methyltransferase^6^,^7^. Severe arteriosclerosis with HHCy was first reported in association with a rare genetic condition in humans, which was also characterized by abnormal cobalamin metabolism and CBS deficiency^8^,^9^. Robinson *et al*.^10^ reported a negative correlation between plasma HCy levels and folic acid levels. Malinow *et al*.^11^ also reported that folic acid, vitamin B_6_ (VB_6_), and vitamin B_12_ (VB_12_) are major determinants of plasma total HCy levels, of which the most close relationship is with folic acid. It is possible that VB_6_ is the coenzyme of CBS and cystathionine-gamma-lyase, catalyzing Met and Serine (Ser) to form cysteine (Cys) and α-ketobutyrate. This is the most important synthetic path for glutathione, which can protect many ingredients of cells against oxidative damage. A number of studies have shown that HHCy has a wide range of biological effects, such as damaging vascular endothelial cells, activating platelets, and stimulating vascular smooth muscle cell proliferation^12^,^13^. HHCy has been strongly correlated with vascular dysfunction. The penis is a richly vascularized sex organ, derived from the spiral artery branch of the iliac arteries and formed in the sponge body of the cavernous sinus. Therefore, we speculated HHCy is caused by vascular endothelial damage, which might affect penile physiologic function. Nitric oxide (NO), a non-adrenergic non-cholinergic (NANC) messenger molecule, is the key element of penile erection. It is proposed that HHCy may affect activity and expression of nitric oxide synthase (NOS). Support for this hypothesis comes from the finding that HHCy inhibits acetylcholine-induced relaxation and cyclic guanosine monophosphate (cGMP) production in the rabbit corpus cavernosum *in vitro*^14^; however, the specific role of HHCy on ED is unclear.

In the present study we established a rat model for HHCy (i.e., >15 μmol/L) through dietary-rich Met in male Sprague-Dawley (SD) rats, and observed the influence of HHCy on reproductive organ weight, sperm density, and total serum testosterone. We also determined the level of expression of two kinds of NOS in rat corpus cavernosum, and evaluated changes in the above indices after administering folic acid, VB_6_, and VB_12_. This study provides experimental basis for the prevention and treatment in male reproductive system disease due to HHCy.

## Results

### Serum testosterone and plasma total HCy level

**Figure 1:**
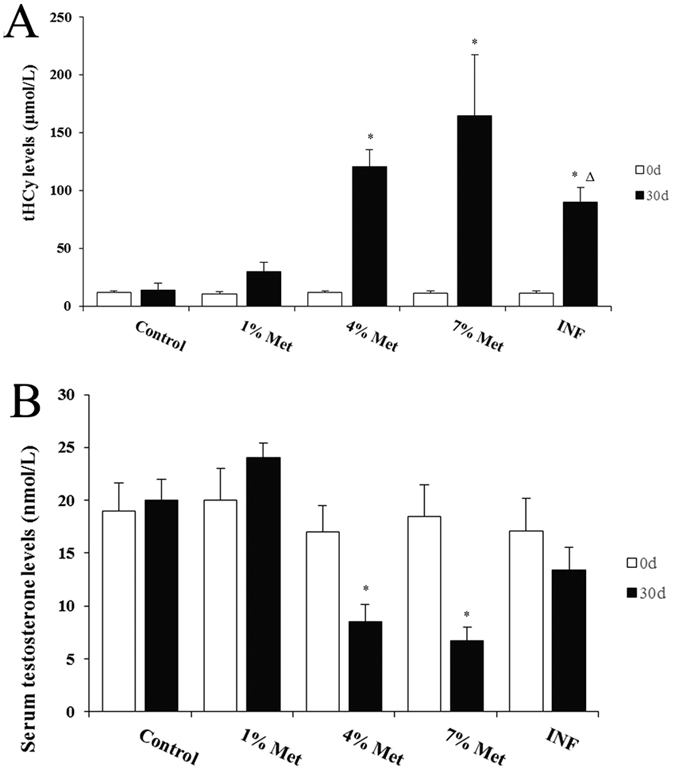
Plasma tHCy levels and serum testosterone levels in all rats. Data are the mean (±standard deviation) of six rats (n = 6 per group, *compared to the control, *P* value < 0.01; Δ compared to the middle-dose group, *P* value < 0.05).

Before the experiments, plasma levels of total HCy (tHCy) were similar in all groups. Interestingly, tHCy of animals fed a diet containing 4%, 7% Met, and the interference group (INF group) for 30 days were significantly higher than the control rats fed a standard diet (mean = 120.61 μmol/L in the middle-dose group, 164.70 μmol/L in the high-dose group, 89.97 μmol/L in the INF group, and 13.74 μmol/L in the controls, *P* < 0.01). We failed to detect a difference in the low-dose group compared with the control group. In addition, total plasma HCy levels in the INF group were markedly lower than the middle-dose group (Fig. 1A). The serum testosterone levels in the middle- and high-dose groups were significantly lower than the control group, unlike the low-dose and INF groups, which was consistent with the relative sperm number (Fig. 1B and Supplementary Figure S1). Taken together, the Met-rich diet led to early and sustained HHCy in an established rat model of HHCy and was consistent with previous reports^12^,^15^.

### Intracavernous pressure measurements and the apomorphine test

**Figure 2:**
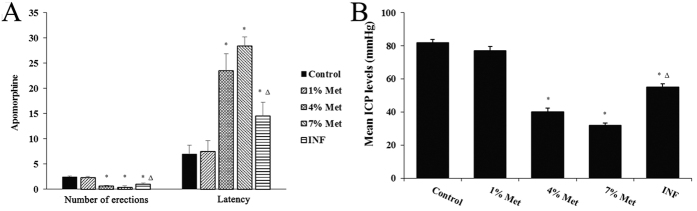
Intracavernous pressure measurement and apomorphine in all rats. Data are the mean (±standard deviation) of six rats (n = 6 per group, *compared to the control, *P* value < 0.01; Δ compared to the middle-dose group, *P* value < 0.05).

In the beginning of the Met-rich diet there were no statistically significant differences in the apomorphine experiments; no significant differences in the number of erections or latency were recorded. At the completion of the 30-day Met-rich diet, the number of erections was significantly lower in the middle- and high-dose groups, and INF group compared to the control group. Furthermore, the latency in the middle- and high-dose groups, and the INF group increased compared with the control group. Moreover, the number of erections in the INF group was increased and latency in the INF group was decreased compared with the middle-dose group. Finally, no significant differences in the number of erections and latency existed between the low-dose and control groups in the experiment (Fig. 2A and Supplementary Table S1). In addition, the intracavernous pressure (ICP) measurement was largely consistent with the results of apomorphine experiments (Fig. 2B). The current study showed that HHCy affected penile erectile function of rats. In general, the number of erections and latency were markedly affected by tHCy levels in a rat model of HHCy.

### Evaluation index of erection

We also showed that three types of hormones were similar in all groups at the start of the experiments. No significant differences were found between luteinizing hormone (LH), follicle-stimulating hormone (FSH), and others parameters, including weight, testis weight, epididymis weight, testis index, and epididymis index, in all rats fed a diet for 30 days compared with the control rats fed a standard diet (Supplementary Table S2).

### Hematoxylin and eosin staining

Hematoxylin and eosin staining (HE) of the cavernous tissue showed that rats fed a Met-rich diet in the middle- and high-dose groups, and INF groups did not affect the histological structure of the tissue despite the poor number of erection and latency compared with the control group (Supplementary Figure S2).

### Immunohistochemical staining and score

**Figure 3:**
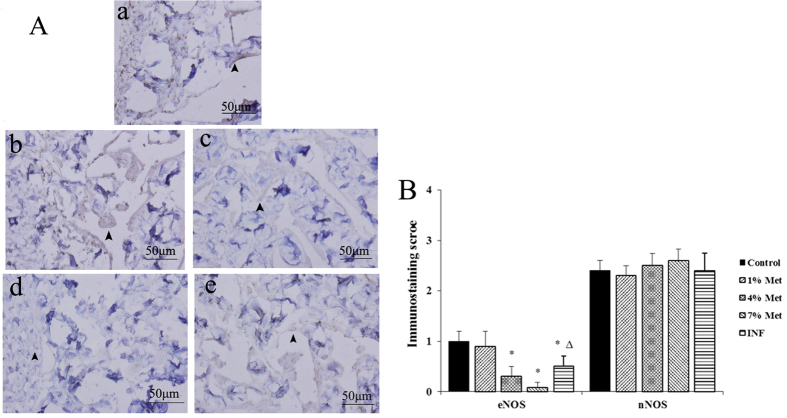
eNOS expression and localization in the penile tissue and immunological score for eNOS and nNOS. (**A**) eNOS immunohistochemical staining. (a) Control group. (b) Low-dose group. (c) Middle-dose group. (d) High-dose group. (e) INF group. The small arrow indicates is protein expression of eNOS (brown–yellow), which is mostly located in the inner wall of the cavernous sinus and blood vessels. (**B**) Immunohistochemical staining score of eNOS. Data are the mean (±standard deviation) of six rats (n = 6 per group, *compared to the control, *P* value < 0.01; Δ compared to the middle-dose group, *P* value < 0.05).

Immunohistochemical analyses showed that endothelial NOS (eNOS), which was located in the inner wall of the cavernous sinus and vessels inner wall, was abundantly undifferentiated in the control and low-dose groups. The quantity of eNOS in the middle- and high-dose groups, and INF group were markedly lower than the control group. Furthermore, eNOS was significantly elevated in the INF group compared with the middle-dose group. No significant differences in the quantity of neuronal NOS (nNOS) existed in the five groups, despite abundant expression of nNOS compared to eNOS in the corpus cavernosum of rats (Fig. 3 and Supplementary Figure S3).

### Reverse transcription polymerase chain reaction and semi-quantitative analysis

**Figure 4:**
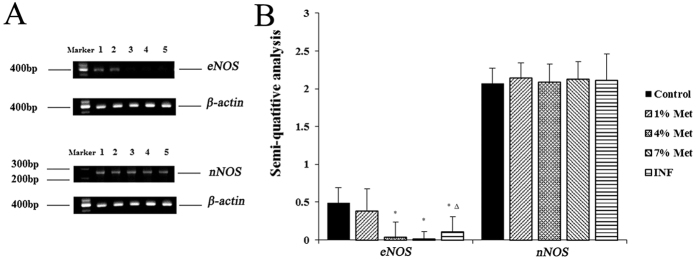
RT-PCR and semi-quantitative analysis of eNOS and nNOS. (**A**) RT-PCR. 1. Control group. 2. Low-dose group. 3. Middle-dose group. 4. High-dose group. 5. INF group. The reference substance is *β-actin*; (**B**) semi-quantitative analysis. Data are the mean (±standard deviation) of six rats (n = 6 per group, *compared to the control, *P* value < 0.01; Δ compared to the middle-dose group, *P* value < 0.05).

Because HHCy induced low levels of eNOS in the middle- and high-dose groups, and INF group, we determined whether or not HHCy affects expression or stability of eNOS in the corpus cavernosum of these rats. We extracted eNOS and nNOS mRNA from the corpus cavernosum of rats using reverse transcription-polymerase chain reaction (RT-PCR). Consistent with other groups examined in this study, the expression of eNOS was decreased. In addition, in the INF group it was increased compared to the middle-dose group, but there was no difference between the INF group and the low-dose or the control groups. There was no significant differences in the expression of nNOS among the groups (Fig. 4).

### Measurement of oxidative stress

**Figure 5:**
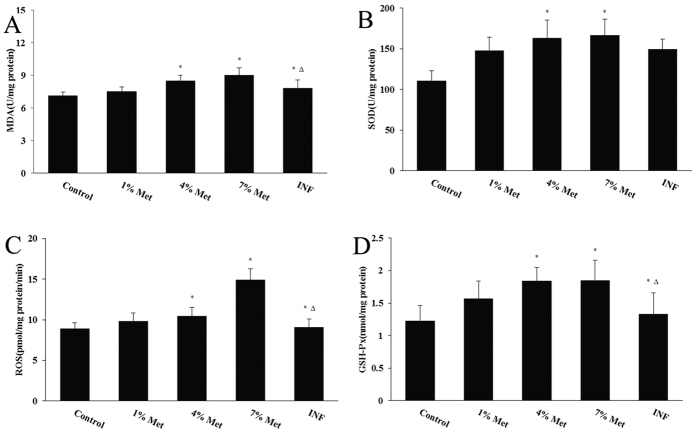
Oxidative stress parameters levels. (**A**) MDA. (**B**) SOD. (**C**) ROS. (**D**) GSH-Px. Data are the mean (±standard deviation) of six rats (n = 6 per group, *compared to the control, *P* value < 0.01; Δ compared to the middle-dose group, *P* value < 0.05).

HHCy suppressed superoxide dismutase (SOD) and glutathione peroxidase (GSH-Px) activity and up-regulated the production of reactive oxygen species (ROS) and malondialdehyde (MDA) in the middle- and high-dose groups, and the INF group compared with the control group (*P* < 0.05). The INF group, and intake of VB_6_ and VB_12_ with folic acid significantly reduced levels of ROS and MDA and enhanced the activity of SOD and GSH-Px compared with the middle-dose group (*P* < 0.05; Fig. 5).

### Western blot measurement of the ratio of phospho-eNOS-to-eNOS

**Figure 6:**
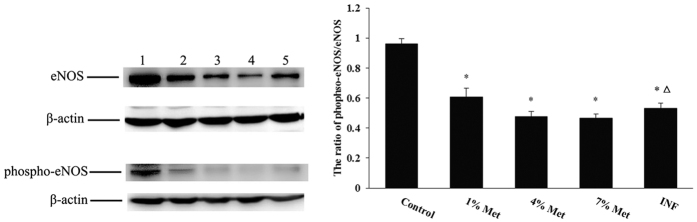
eNOS and phospho-eNOS protein expression in the penile tissue and the ratio of phospho-eNOS/eNOS. (**A**) Western blot. 1. Control group. 2. Low-dose group. 3. Middle-dose group. 4. High-dose group. 5. INF group. The reference substance is β-actin; (**B**). The ratio of phospho-eNOS/eNOS. Data are the mean (±standard deviation) of six rats (n = 6 per group, *compared to the control, *P* value < 0.01; Δ compared to the middle-dose group *P* value < 0.05).

We found that the ratio of phospho-eNOS-to-eNOS in the low-, middle-, and high-dose groups, and the INF group was significantly lower than the control group (*P* < 0.05). Moreover, the ratio of phospho-eNOS-to-eNOS in the INF group, and intake of VB_6_ and VB_12_ with folic acid was significantly increased compared with the middle-dose group (*P* < 0.05; Fig. 6).

## Discussion

ED is a common medical disorder, affects 35–65% of males >50 years and it has a negative impact on the quality of life of millions of males worldwide^16^,^17^. The penis is a richly vascularized organ and ED is predominately a vascular disease. There are numerous causes of vasculogenic ED, including hypertension, DM, coronary artery disease, peripheral vascular disease, and smoking^18^,^19^. Additionally, HHCy is a recognized risk factor for endothelial dysfunction and related disorders, including CVD and ED^20^,^21^. NO, the key mediator synthesized by different NO synthase isoenzymes (eNOS, nNOS, and inducible NOS), plays an essential role in endothelial function^22^,^23^,^24^. Sexual stimuli induce an increase in serum levels of testosterone and the release of NO from penile nerve endings and endothelial cells, which in turn relax cavernous smooth muscle and increase blood flow to the penis^25^. It has been demonstrated that HHCy impairs erectile function by impairing NO release from endothelial NO synthase by the endogenous overproduction of O^2−^, and the intake of VB (especially folate, VB_6_, and VB_12_) slightly decreases symptoms of ED^9^,^26^.

ED is highly prevalent, affecting at least 50% of males with DM^27^,^28^,^29^. In patients with diabetes, some of the physiologic mechanisms are the result of compromises, which induce ED as a “side effect”. In diabetes, hyperglycemia is associated with increased oxidative stress due to an overproduction of advanced glycation end products, hexosamine, and protein kinase C, and increased stimulation of the polyol pathway^30^,^31^. Some studies^20^,^27^,^31^ have reported that insulin regulates the liver function, and in turn sulfonium enzyme activity regulates blood HCy concentration, leading to a drop in blood HCy levels. It is believed that there is a lack of insulin or insulin resistance in patients with DM, and abnormal insulin action may affect the catabolism of HCy and cause HHCy, thus exposing diabetic patients to further risk for the development of ED^32^,^33^. A negative correlation exists between insulin and plasma HCy levels^20^.

In the present study the methionine-rich diet led to HHCy in an established rat model of HHCy. We showed that HHCy existed in all treatment groups, but the plasma total HCy concentration in animals in the middle- and high-dose groups, and the INF group, were significantly higher than the control rats fed a standard diet, suggesting that a Met-rich diet might induce HHCy. Furthermore, plasma levels of total HCy in the INF group were markedly lower than the middle-dose diet group without accompanying significant changes in the plasma levels of LH or FSH in all groups, as reported previously^34^. It is therefore suggested that intake of VB may have a beneficial effect against HHCy and it should be viewed in relation to therapeutic methods.

Apomorphine is a synthetic morphine derivative and a short-acting dopamine receptor agonist, which induces centrally-mediated erectile responses without narcotic or analgesic effects, and apomorphine-induced erection is an available and simple method^25^,^35^,^36^. When a small dose of apomorphine was injected into the rats, erectile events were produced. Several previous studies have used apomorphine to investigate erectile responses in animal models^21^,^35^,^37^,^38^. Erectile responses after apomorphine injection are very rapid. The findings of the present study are particularly relevant, taking into consideration that the number of erections in the middle- and high-dose groups, and the INF group was lower than the control group, and latency in the middle- and high-dose groups and INF group increased compared with the control groups after 30 days of a Met-enriched diet, suggesting that HHCy was a significant risk for erection in a rat model. In addition, the ICP measurement was largely consistent with the results of the apomorphine experiments.

It is well-known that testosterone acts on the central and peripheral systems to help regulate libido and erectile function^30^,^39^,^40^. We found that serum testosterone levels in the middle- and high-dose groups were significantly lower than the control group, but not different than the low-dose and INF groups with similar sperm counts, suggesting that HHCy affected libido, and erection^34^,^41^. No significant differences existed between LH and FSH levels, including weight, testis weight, epididymis weight, testis index, and epididymis index in all rats fed a diet for 30 days compared with the control rats fed a standard diet. HE staining of the corpus cavernous tissue showed that rats fed Met-rich diet in the middle- and high-dose groups and the INF group did not affect histology, despite the poor number of erections and latency compared with controls. HHCy did not influence the structure of the genital tract, such as the testis, epididymis, and penis, in our rat model.

Prospective studies have indicated that HHCy is an important regulator of NO synthase^15^,^20^. One feature of HCy is that it affects NO formation and is markedly augmented by copper, which is an independent risk factor for developing vasculopathy^42^. In isolated rabbit aorta and cavernosum, copper augments the inhibitory effects of HCy on endothelium-dependent relaxation *in vitro*^29^,^42^. HCy has also been shown to have a greater negative effect on endothelial NO in an experimental rabbit model of DM, which is associated with increased plasma copper and HCy levels^29^. These interactive effects were shown to be mediated by an augmentation of O^2−^ formation.

In immunohistochemical analysis of eNOS and nNOS, we found that the quantity of eNOS in the middle- and high-dose groups, and the INF group was markedly decreased compared to the control groups, and significantly elevated in the INF group compared with the middle-dose group. No significant differences in nNOS existed in the five groups, despite more abundant expression of nNOS than eNOS in rat corpus cavernosum. Thus, HHCy effects release of eNOS in rat corpus cavernosum, but not nNOS. The expression of eNOS decreased in the middle- and high-dose groups, as well as the INF group compared with the control group. In addition, eNOS was increased more in the INF group than the middle-dose group, but there was no difference between the low-dose and control groups. No difference was detected in the expression of nNOS in all groups. Thus, it is reasonable to suggest that HHCy affects the expression of eNOS in the corpus cavernosum of these rats, but not nNOS.

Protein levels of eNOS and phospho-eNOS in the penile tissue as well as the ratio of phospho-eNOS-to-eNOS were significantly decreased in the middle-dose, high-dose, and INF groups compared to the control group. On the other hand, these proteins expressions and the ratio were significantly increased in the INF group than in the middle-dose group. It appears that HHCy inhibited the eNOS and phospho-eNOS protein expressions and HHCy is a vascular risk factor for erectile dysfunction by inducing endothelial dysfunction. From the present data VB may provide a helpful tool for the decrease of ED in HHCy patients.

The present data on the oxidative stress parameters indicate that HHCy suppress the anti-oxidant defenses of the penile tissue and induces up-regulation of ROS and lipid peroxidation. Intake of VB_6_, VB_12_ and folic and folic acid in the INF group significantly decreased oxidative stress production and enhanced the antioxidant protective mechanism of the tissue by increasing the levels of antioxidant enzymes. To our knowledge this is the first time in the international literature that HHCy-induced oxidative stress in the penile tissue is negatively connected with the mitigated eNOS mRNA and protein levels in the penis. Decrease of the oxidative stress resulted in the improvement of eNOS expressions in the penis from INF animals. These results underline the importance of antioxidant agents in the alleviation of ED^43^,^44^,^45^.

Males with DM and ED are considered one of the most difficult to treat subgroups. Phosphodiesterase type 5 inhibitors, a pharmaceutical agent with anti-oxidant properties, are the first-line treatment option for ED, although the efficacy is lower compared to the general ED population^28^,^46^,^47^. Numerous prospective studies have reported that patients with ED who have long-term intake of folic acid and vitamin B_12_ can reduce the blood concentration of HCy and delay the onset of HHCy^26^,^31^,^48^. Satisfactory results were obtained in patients with combined therapy of sildenafil, folic acid, and VB_6_, through which plasma HCy was reduced^26^.

In the present study HHCy impaired erectile function in rats by decreasing eNOS from the corpus cavernosum of the penis. This result demonstrated that HHCy might be a vascular risk factor for ED by impairing cavernosa endothelial NO synthase activity. A limitation of this study includes the comparatively small number of animals per group (n = 6). This resulted into a rather higher standard deviation. Further experimental and epidemiological data are required to advance our knowledge on the impact of HHCy on erectile function. The findings will contribute to therapy of patients with ED.

To summarize, a Met-rich diet for 4 weeks led to HHCy in rats, and supplemental VB helped relieve the symptoms and was associated with inhibition of endothelium-dependent relaxation and NO-mediated cGMP formation in the isolated corpus cavernosum, an effect mediated by eNOS. This study provides further support for the hypothesis that HHCy represents a vascular risk factor for ED by impairing cavernosa endothelial NO synthase activity. Further prospective studies in patients with ED are required to determine whether or not these observations are of clinical relevance, and in particular whether or not there is a causal link between HCy and ED.

## Methods

### Animal care

This project was carried out with the approval of the Academic Administration Committee of Jinling Hospital at Nanjing University School of Medicine. All animal experiments were conducted in accordance with the guidelines of the Animal Ethical Committee for Animal Experiments in China, which are consistent with the US National Institutes of Health guidelines. Thirty male SD rats (stock #2014070615), 10 weeks of age and weighing 315–340 g, were provided by the Department of Comparative Medicine of Jinling Hospital at Nanjing University School of Medicine in Nanjing, and were individually housed in a conventional animal facility with laminar flow maintained at 20 ± 1 °C and 50 ± 10% relative humidity with a 12 h light:12 h dark photoperiod. Food and water were provided *ad libitum*. After a 1-week acclimatization period with standard rat chow, the rats were randomly divided into control, low-dose (1% Met), middle-dose (4% Met), high-dose (7% Met), and INF groups (4% Met; n = 6 per group), in which folic acid (0.5 mg), VB_6_ (2.5 mg), and VB_12_ (50 μg) were given orally to the rats daily for 1 month. During the experiments, all rats were weighed weekly.

### Blood biochemical tests

At baseline and day 30, blood was collected from the retro-orbital plexus in a capillary tube, mixed with an anticoagulant (2% ethylene diamine tetraacetic acid calcium disodium [EDTA-Na_2_, 1 mL/mL]), rapidly frozen, and stored at −20 °C until analyzed. The total plasma HCy level were measured with an AXSYM Automatic immunity analyzer (Abbott, Inc., Chicago, IL, USA) by a competitive immunoassay using the AXSYM Homocysteine kit in accordance with the manufacturer’s instructions. At baseline and day 30, plasma samples were obtained by centrifugation at 3000 g for 10 min and stored at −20 °C until used. The testosterone concentration in serum was measured using a radioimmunoassay method in accordance with the manual for the Access Testosterone kit (Beckman Coulter Inc., Los Angeles, CA, USA).

### Apomorphine experiments and ICP measurement

At the start of the experiment (0 day) and day 30, we carried out apomorphine experiments on rats in a dark room with sound insulation. Apomorphine (Sigma, St. Louis, MO, USA) was prepared with 0.1% ascorbic acid and injected subcutaneously into the nape of the neck at a dose of 80 μg/kg after a 1-h adaptive period in an indoor environment. After the injection, the rats were immediately placed into a transparent Plexiglas cage (30 × 25 × 25 cm), reliably observed, and the number of penile erections and latency within 30 min were recorded. A penile erection was considered to occur when the following behaviors were present: the animal stood upright; bent its head down to reach the genital area; held its engorged penis by the forepaws; and licked the glans penis. A wide opening of the mouth with associated appropriate respiratory movement identified a yawn. We measured the latency of the first erection and the number of erections and yawns per animal. Latency was recorded as 30 min if no erection occurred during the observation period^34^,^41^.

Thirty days after a Met-rich diet, rats were anesthetized with an intraperitoneal injection of pentobarbital (200 mg/kg; Heng Rui Pharmaceutical Inc., Lianyungang, Jiangsu, China) and fixed in the supine position. We then performed a midline incision of the lower abdomen. The major pelvic ganglia and cavernous nerves were exposed using a surgical microscope (×10 magnification). A bipolar electrode connected to an electrical pulse stimulator was placed onto the cavernous nerves. Next, an additional oblique incision was performed to allow dissection and exposure of the corpora penis^33^,^34^. A 23-gauge butterfly needle connected to a pressure recorder (to allow recording of the ICP value) was then inserted into the crus penis. Before needle insertion, sterilized heparin (100 U heparin/mL) was aspirated into the needle to prevent occlusion due to blood coagulation. The cavernous nerves were then stimulated by sustained electrical pulses with a pulse width of 2 ms, a frequency of 20 Hz, and an intensity of 5 V. A single stimulation lasted for a total of 60 s, and the interval between two single stimulations was 2–3 min. Three stimulations were conducted per animal, and the maximal amplitude of ICP during nerve electrostimulation was calculated from the baseline value and included for statistical analysis into each animal. After functional testing, rats were euthanized by intraperitoneal injection of pentobarbital (200 mg/kg), following with bilateral thoracotomy^35^,^36^,^37^. Some tissues were then harvested for histologic analysis.

### Tissue preparation

At the final stage of the apomorphine experiments, the rats were sacrificed and animals were euthanized by intraperitoneal injection of pentobarbital (200 mg/kg), following with adhesive connective tissue-free testes and epididymes being dissected off to store on ice. Then, we weighed the testes and epididymes using an electronic balance, and calculated the organ coefficient of the testis according to the following formula: 100 × weight of testis divided by body weight (units are mg/100 g). Similarly, we weighed the epididymis using an electronic balance, picked up the cauda epididymidis according to the anatomic localization, and placed in the six holes in the cell culture plate. Based on the method described by Jalili *et al*.^48^, we added 2.0 mL of saline, cut the cauda epididymidis in the middle into two segments with ophthalmic scissors, shook well for 30 min (50 g removed the supernatant, and mixed with distilled water (1:20), dripped into the blood count, and counted by conventional methods after standing for 3 min. The relative sperm relative count (10000/100 mg of epididymis weight) was calculated according to the following formula: 100 × sperm count divided by epididymis weight.

### HE and immunohistochemistry

Rats were euthanized by intraperitoneal injection of pentobarbital (200 mg/kg) given via the lateral ear vein after the apomorphine experiments. The penis with a size of 1 × 3 cm was harvested and the corpus cavernosal tissues were dissected from the tunica albuginea and fixed in 4% paraformaldehyde solution at 4 °C overnight^49^. One sample of tissue was obtained from each specimen and blocked with paraffin for routine follow-up procedures. Consecutive sections with a thickness of 4 μm were prepared. The region of interest of the penis underwent HE staining and immunohistochemistry staining.

The primary antibodies (anti-eNOS mouse anti-rat and anti-nNOS mouse anti-rat; BD Transduction Laboratories, Inc., Franklin Lakes, NJ, USA) were diluted 100 times using phosphate-buffered saline (PBS) and the specimens were bathed overnight. The secondary antibodies (immunoglobulin [IgG]/horseradish peroxidase goat anti-mouse; KPL, Inc., Hemet, CA, USA) were diluted 800 times using PBS and the specimens were soaked in PBS three times for 30 min each cycle. Then, all specimens soaked in a color agent (DAB-H_2_O_2_; Boster, Wuhan, China) for 10 min. The sections were washed with running tap water before and after each solution, dehydrated in an alcohol series, and mounted with gum. The results were observed and photographed under microscopy with a 100-fold amplification.

### Measurement of oxidative stress

The activities of GSH-Px and SOD and the amount of MDA and ROS were measured using commercially available detection kits according to the manufacturers’ instructions (Nanjing Jiancheng Bioengineering Institute, Nanjing, China). The samples from the penile tissues of rats were analyzed with a SpectraMax M2 spectrometer (Molecular Devices, Sunnyvale, CA, USA). The activity of SOD was expressed as U/mg protein (λ = 450 nm). The activity of GSH-Px was expressed as U/mg protein (λ = 412 nm). The level of ROS was expressed as pmol/mg protein/min (λex = 488 nm, λem = 520 nm). The level of MDA was expressed as nmol/mg protein (λ = 523 nm).

### Immunohistochemical staining results in a semi-quantitative score

All samples were categorized based on the extent of immunoreactivity, as follows: 0, <5%; 1, 5–10%; 2, 10–50%; and 3, >50%. Staining intensity was scored as follows: 0, negative; 1, weak; 2, moderate; and 3, strong. For each case, the total immunostaining score, also known as the staining index, was calculated by multiplying the percentage of positive cells with the staining intensity score, yielding a value between 0 and 3.

### Reverse transcription- polymerase chain reaction (RT-PCR)

Total RNA was extracted from rat penis tissues using TRIzol reagent (Invitrogen, Carlsbad, CA USA) by following the manufacturer’s instructions. Then, the RNA was quantified by using a spectrophotometer. All RNA samples with an A_260_/A_280_ of 1.8–2.2 were used for reverse transcription. The extracted total RNA (2 μL [1 μg]) was employed for first-strand cDNA synthesis using 20 μL of random primers at 42 °C for 30 min and 95 °C for 5 min. cDNA from the reverse transcription reaction was then used in the subsequent PCRs with eNOS, nNOS, and *β-actin* primers. The primer sequences were designed by GeneCore BioTechnologies Co. (Shanghai, China; Supplementary Table S3). For a reaction volume of 25 μL, 0.5 μL of 10 mM dNTPs, 5 μL of 5 × Taq buffer, 2.5 μL of 25 mM MgCl_2_, 1 μL of 1 μM primer, 2 μL of cDNA, 12.875 μL of DEPC water, and 0.125 μL of Taq enzyme (Promega, Madison, WI, USA. 25 U/μL) were added to the reaction mix. The reaction conditions were as follows: 95 °C for 5 min; 30 cycles of 95 °C for 40 s; 53 °C for 30 s; and 72 °C for 40 s, followed by 72 °C for 7 min, and stored at 4 °C. The mRNA of β-actin was used as the internal control probe.

We mixed 5 μL of PCR products and 1 μL of bromophenol blue, and analyzed in a 1.5% agarose gel with electrophoresis, using an ultraviolet gel image analysis system photograph and BandScan software (Nanjing my god biotechnology company limited, Nanjing, China) analysis. The relative expression quantity of mRNA of the target gene was the ratio of relative optical density value of the target gene and optical density value of *β-actin*.

### Total eNOS and phospho-eNOS

To examine the ratio of phospho-eNOS-to-eNOS, western blots were performed by collecting the penile tissues from control and Met-rich diet rats. The same amount of protein homogenate of all groups were exposed to sodium dodecyl sulfate polyacrylamide gel electrophoresis and analyzed through Western blot processes with anti-human/mouse/rat primary antibodies (all 1:800, overnight at 4 °C), including total eNOS, phospho-eNOS, and β-actin (all Bioword Technology, Inc., Louis Park, MN, USA). After a 1-h incubation at room temperature with goat anti-rabbit IgG secondary antibody (1:10000; Bioword Technology, Inc.), all proteins were identified by Biodlight ECL Chemiluminescent HRP Substrate (Bioword Technology, Inc.). A gel imaging analysis system (Tanon Inc., Shanghai, China) was used to quantify the protein bands expressed as a ratio.

### Statistical analyses

All data are presented as the mean ± standard deviation. The data were analyzed with one-way analysis of variance, followed by the least significant difference test for *post hoc* comparison with statistical significance accepted at a *P* < 0.05. All the analyses were performed using SPSS 15.0 software (SPSS, Inc., Chicago, IL, USA).

## Additional Information

**How to cite this article**: Jiang, W. *et al*. Hyperhomocysteinaemia in rats is associated with erectile dysfunction by impairing endothelial nitric oxide synthase activity. *Sci. Rep.* **6**, 26647; doi: 10.1038/srep26647 (2016).

## Supplementary Material

Supplementary Information
