# Supplementary material for: Hyperhomocysteinaemia in rats is associated with erectile dysfunction by impairing endothelial nitric oxide synthase activity
Source: Sci Rep. 2016 May 25;6:26647. doi: 10.1038/srep26647 (PMC4879575; doi:10.1038/srep26647)
Supplement: Supplementary Information [file srep26647-s1.pdf]

Hyperhomocysteinaemia in rats is associated with erectile dysfunction by impairing endothelial nitric oxide synthase activity

Weijun Jiang[1], Lei Xiong[2], Bin Yang[1], Weiwei Li[1], Jing Zhang[1], Qing Zhou[1], Qiuyue Wu[1], Tianfu Li[1], Cui Zhang[1], Mingchao Zhang[1] and Xinyi Xia*[1]

1 Department of Reproduction and Genetics, Institute of Laboratory Medicine, Jinling Hospital, Nanjing University School of Medicine, Nanjing 210002, P.R. China

2 Department of Cardiothoracic surgery, Jinling Hospital, Nanjing University School of Medicine, Nanjing 210002, P.R. China

Email addresses:

WJJ: 1048281756@qq.com

LX: threestone1983@163.com

BY: ybdz@163.com

WWL: eeeeet@sina.com

JZ: 710081649@qq.com

QZ: 863840726@qq.com

QYW: 475626760@qq.com

TFL: talentli13@163.com

CZ: 841479480@qq.com

MCZ: zmchj99@163.com

*XYX: xiaxynju@163.com

Correspondence: Xinyi Xia, Department of Reproduction and Genetics, Institute of Laboratory Medicine, Jinling Hospital, Nanjing University School of Medicine, Nanjing, P.R. China

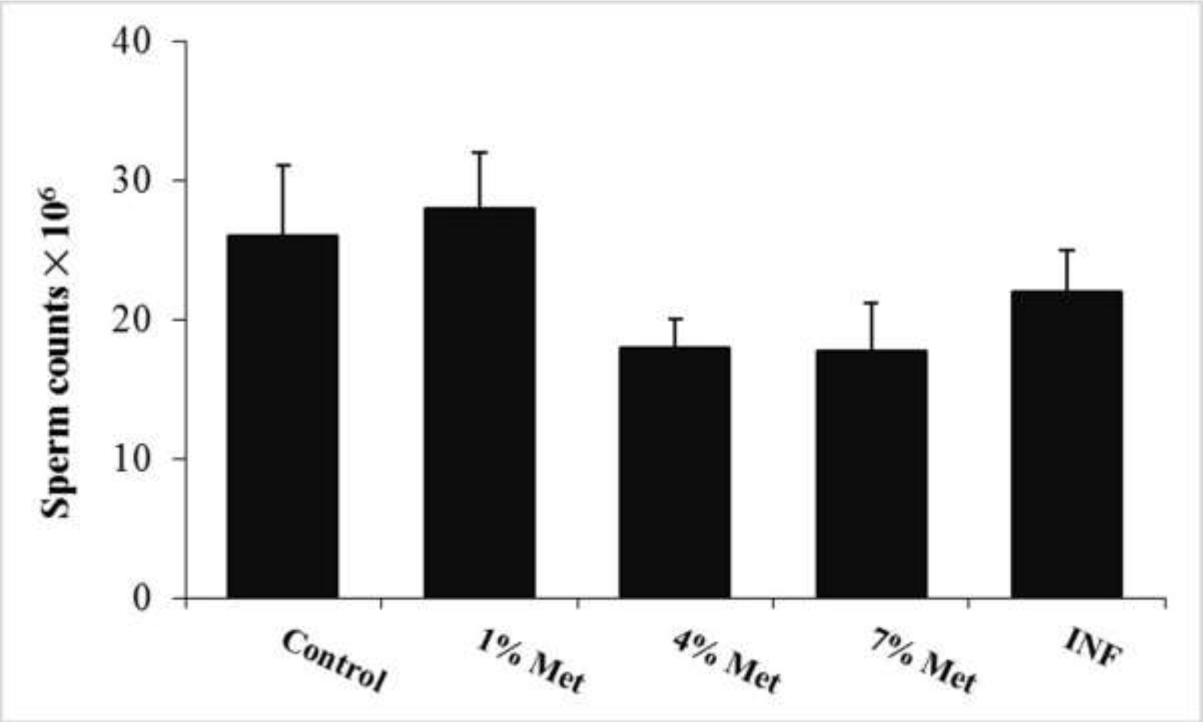

Supplementary Figure S1. Sperm number of five groups of rats. n = 6 per group.

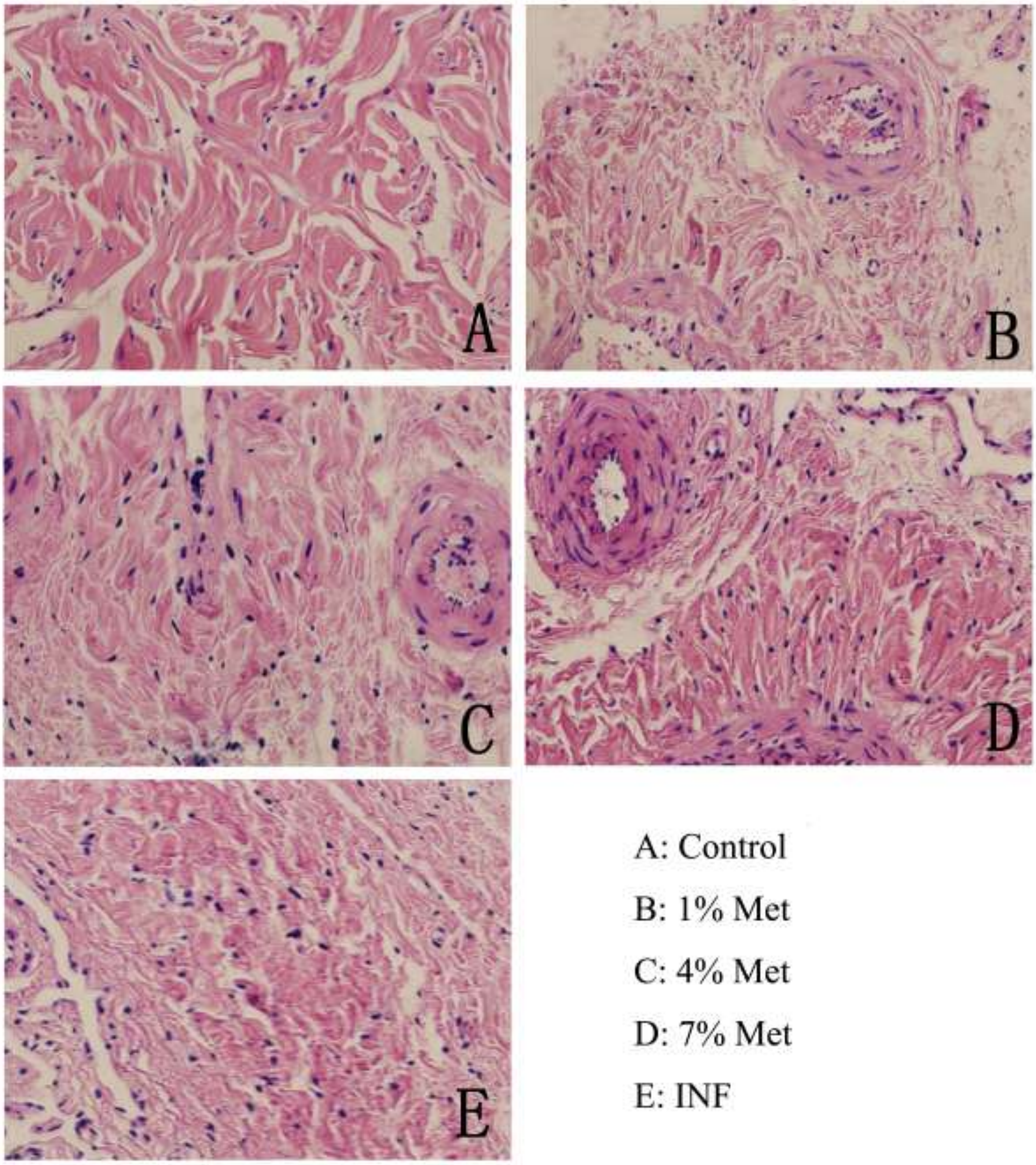

Supplementary Figure S2. Penile corpus cavernosal tissue of rats stained with hematoxylin and eosin. A. Control group. **B**. Low-dose group. **C**. Middle-dose group. **D**. High-dose group. **E**. INF group. Sections were made transversal to the penile axis. HE staining of the penile corpus cavernosal tissue showed that all rats did not affect organization structure. (Original magnification ×320; scale bar = 50 μm).

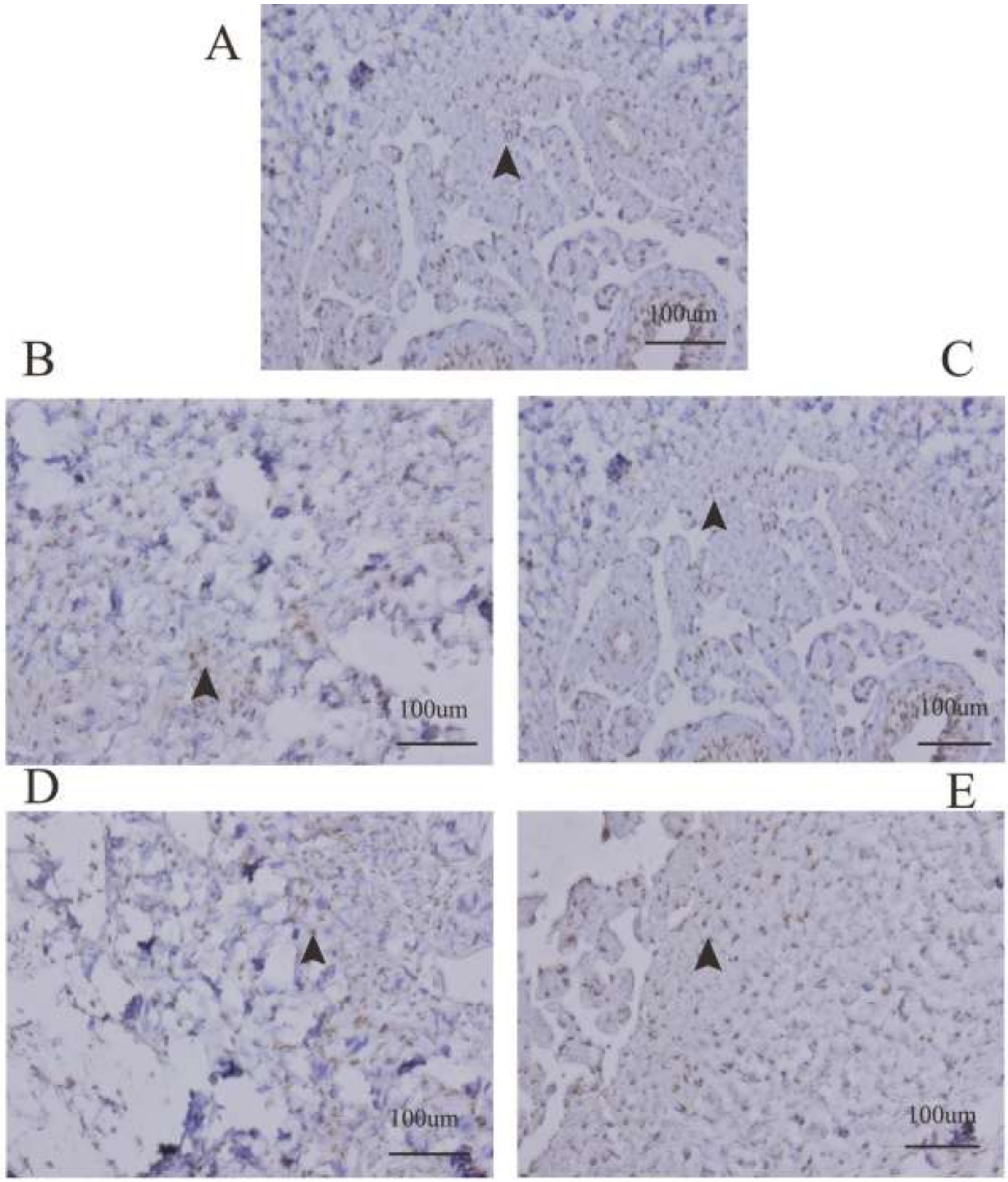

Supplementary Figure S3. Protein expression of nNOS in cavernous tissue. A. Control group. **B**. Low-dose group. **C**. Middle-dose group. **D**. High-dose group. **E**. INF group. The small arrow indicates protein expression of nNOS (brown-yellow), which is abundantly distributed in the inner wall of the cavernous sinus.

Supplementary Table S1. The result of apomorphine experiment

Parameter (min)	Control	1% Met	4% Met	7% Met	INF
Numbers of erection (0 d)	2.34±0.34	2.45±0.66	2.26±0.67	2.44±0.42	2.38±0.23
Erectile Latency (0 d)	7.56±2.22	6.88±3.45	7.89±3.01	8.05±3.46	6.94±3.26
Numbers of erection (30th d)	2.40±0.26	2.33±0.22	**0.60±0.13***	**0.36±0.29***	**1.01±0.25***$^{\Delta}$
Erectile Latency (30th d)	6.96±1.77	7.46±2.20	**23.46±3.42***	**28.37±1.88***	**14.49±2.77***$^{\Delta}$

* compared to the control, *P* value < 0.01; Δ compared to the middle-dose group *P* value < 0.05. Bold Font indicated the group exist in significantly difference.

Supplementary Table S2. Weight and index of testis and epididymis of all rats

Group	Weight(g)	Testis weight(mg)	Epididymis weight(mg)	Testis index(mg/g)	Epididymis index(mg/g)
Control	507.6±58.3	1648.5±317.5	609±29.0	327.2±41.8	130.8±29.1
1% Met	525.8±35.2	1857.5±112.4	710.0±56.6	354.7±37.7	147.8±26.7
4% Met	465.4±32.9	1509.0±219.2	550.0±66.5	334.5±15.6	123.9±18.7
7% Met	466.2±19.5	1537.0±113.1	528.5±58.9	314.9±39.9	118.6±39.9
INF group	481.2±25.9	1551±134.6	557.5±38.2	349.5±97.0	125.3±37.7

Data are the mean±standard deviation of six rats. (n = 6 per group)

Supplementary Table S2. The primer information of eNOS, nNOS and β-actin.

Primers name	Forward (5'--3')	Reverse (5'--3')	Length (bp)
eNOS	GGTGAACAAGGAGATTGAAAGCA	CACACTCGGAAGTCATGTTTGC	361
nNOS	CTCGAGCGGTGGACACAAG	TCCAGACGCACCAGGATTG	276
β-actin	AGCTGTGCTATGTTGCCCTAGAC	GCCAGGATAGAGCCACCAATC	397
